# Incomplete description of the current body of evidence of the health economics of Duchenne muscular dystrophy

**DOI:** 10.1186/s13023-018-0975-3

**Published:** 2019-04-02

**Authors:** Erik Landfeldt, Hanns Lochmüller, Peter Lindgren

**Affiliations:** 1 Institute of Environmental Medicine, Karolinska Institutet, Nobels väg 13, SE-17177 Stockholm, Sweden; 2 ICON plc, Stockholm, Sweden; 3 Department of Neuropediatrics and Muscle Disorders, Faculty of Medicine, Medical Centre – University of Freiburg, Freiburg, Germany; 4 Children’s Hospital of Eastern Ontario Research Institute, University of Ottawa, Ottawa, Canada; 5 Division of Neurology, Department of Medicine, The Ottawa Hospital, Ottawa, Canada; 6 Medical Management Centre, Department of Learning, Informatics, Management and Ethics, Karolinska Institutet, Stockholm, Sweden

Recently, Ryder et al. [1] published results from a systematic literature review of the burden, epidemiology, costs, and treatment of Duchenne muscular dystrophy (DMD), a rare, terminal, neuromuscular disease for which several molecules currently are being tested in trials. The review provides a somewhat peculiar perspective of the current body of evidence of these aspects of DMD, in particular the economic burden and patient quality of life, which in our opinion may result in misconceptions of prevailing data gaps concerning evidence employed in health technology assessments. In addition, there are several components of the synthesis and reporting of the identified evidence which warrant clarification to avoid confusion. The purpose of this commentary is to highlight some of the shortcomings in the search strategy and synthesis of cost and quality of life data reported by Ryder et al. that ultimately result in a description that we find is at odds with our understanding of the health economic context of DMD.

First, the systematic literature review by Ryder et al. only considered records published up until June 2015. As a consequence, several recent key publications on the economic burden and patient quality of life were not identified. Examples include our own work [2, 3], as well as the work of others [4–9]. In addition, several studies of costs and quality of life in DMD published between 2005 and June 2015 are not included in the review (e.g. [10–16]). For these reasons, the outcomes of the review provide an incomplete description of the current evidence base.

Second, Ryder et al. claim they only included studies of patients with DMD, not mixed populations of e.g. patients with muscular dystrophy. This is a reasonable criterion for robust evidence synthesis and meaningful comparison, as e.g. Becker muscular dystrophy and DMD have very different pathophysiology and thus impact in terms of burden, costs, and quality of life. However, of the three identified cost studies, one [17] is indeed a study of a mixed population of patients with hereditary progressive muscular dystrophy, which includes diagnosis of DMD but also other forms of muscular dystrophy.

Third, in their synthesis of identified cost data, Ryder et al. employ ambiguous labels for reported subgroups and make several incorrect observations when comparing the data reported in the identified publications. For example, in Table 5 in their article, which according to the table title provides a summary of direct healthcare costs (which also is incorrect as some of the reported data contains direct non-medical costs), country-specific labels for the first stratum from Landfeldt et al. [18] read “DMD Age 9 to 17” which most readers would interpret as the range (i.e. the minimum and maximum patient age) of the sample, when it in fact refers to the inter-quartile range (the range was 5–34 years). Moreover, when contrasting cost evidence from different studies, the authors provide little guidance to help readers understand if the different estimates are indeed comparable (with respect to e.g. included resources and valuation techniques). The authors also employ a categorisation of costs (i.e. direct healthcare costs, indirect costs, social care costs, and out-of-pocket expenses) which is not very helpful, as the identified studies estimate and combine these cost components differently.

Fourth, concerning evidence of patient quality of life, Ryder et al. state that parent proxy scores were similar to patient self-assessments of quality of life. However, no explanation is provided for this claim, which in our experience is not entirely accurate. In fact, we have reported as part of our own research [2] that the level of agreement between patient self-assessments and caregiver proxy-assessments to be poor to fair [10, 19], poor to fair to moderate [11], moderate [15], and moderate to good agreement [2, 20]. With respect to the synthesis of the quality of life data, it is unfortunate that the authors devote little effort to contextualize current evidence with respect to e.g. evidence of coping mechanism, self-perceived versus objective quality of life (i.e. as assessed through general population preferences), issues concerning the measurement of quality of life in paediatric populations (which is particular relevant to a genetic childhood disease such as DMD where a non-trivial proportion also suffer from cognitive impairments), and issues concerning precision and generalizability.

Fifth, and last, Ryder et al. claim to assess the quality of the identified records using the STROBE criteria, however, no explanation on how this assessment was conducted is provided. We would suggest authors of future reviews to instead assess the validity of the evidence to facilitate an understanding of e.g. generalizability of results and potential biases, as subjective evaluations based on what can be regarded as “adequate” or “representative” in our experience easily result in misunderstandings.

We hope that this commentary will help readers interpret the review by Ryder et al. and hopefully avoid misguided research initiatives into aspects of the health economics of DMD already described. That being said, there are obviously data gaps concerning costs and quality of life in DMD that warrant further study, although these are unfortunately not correctly mapped out in work by Ryder et al.
