# Supplementary material for: Incomplete description of the current body of evidence of the health economics of Duchenne muscular dystrophy
Source: Orphanet J Rare Dis. 2019 Apr 2;14:75. doi: 10.1186/s13023-018-0975-3 (PMC6446357; doi:10.1186/s13023-018-0975-3)
Supplement: Related texts

# Reply: Author’s response

**Authors:** S. Ryder, R. M. Leadley, N. Armstrong, M. Westwood, S. de Kock, T. Butt, J. Kleijnen

Whilst the commentary by Landfeldt et al. does highlight a couple of deficiencies in the original review, most of the comment is unwarranted or inaccurate. The contents of the commentary do, however, suggest that evidence is rapidly changing and that the period covered by the review up until June 2015 is perhaps not reflective of the findings of more contemporary research including that published by the authors of the letter.

Each of Landfeldt’s five points is addressed in turn as follows:

## Missing studies

Authors of the review explicitly state “The main potential limitation of our approach was in its restriction to published and unpublished evidence from 2011 to 2015”. In this sense Landfeldt and colleagues are agreeing with the authors of the paper. Aside from evidence produced after June 2015, Landfeldt and colleagues suggest that seven other studies have been missed. However, this is factually inaccurate in that four of these studies [10, 11, 13, 16] were published earlier than the time frame mentioned above i.e. 2011 to 2015. There is a misleading phrase in the Ryder et al. [1] manuscript which suggests “we decided to focus on records from 2010 onwards” this could have perhaps been better written “all records between 2011 and June 2015 were the focus of the review”. Landfeldt et al. do point to three additional studies [12, 14, 15]: which in their view should have been included. Reasons for exclusion could be further researched if necessary but ultimately no review process, particularly one which considers both published and unpublished research can claim to be 100% infallible.

## Study with an incorrect population

Landfeldt et al. suggest that one of the included studies [17] relates to a population which is not restricted to DMD only (it is described as a proxy for DMD). This point is correct. However, the study does, it could be argued, provide useful international comparative data albeit on a contentious definition of DMD. The study was assessed as low quality in the Ryder et al. review which suggests caution of interpretation in any case.

## Ambiguous subgroups and incorrect observations

The Landfeldt letter suggests that Ryder et al. use ambiguous labels for subgroups and incorrect observations. The example they suggest is Table 5 which they suggest is incorrect because direct healthcare costs include direct non-medical costs. This is merely opinion. Healthcare is broader than just medical. For example, physiotherapy and occupational therapy is not “medical” expenditure but is “healthcare” expenditure. We would agree that there are different categorisations of cost and that researchers estimate and combine costs differently but this is very much a feature of the literature and not just the review by Ryder et al. Landfeldt et al. will be well aware of differences in cost categorisation used by different authors and that it is difficult/impossible to assess comparability (particularly between countries). It would be great to be more prescriptive but this may not be possible. Landfeldt points out that the range referred to in Landfeldt et al. 2014 is an IQR (and indeed this could have been made more explicit in the paper by Ryder et al. [1]).

## Parent proxy and self-assessment

The comments in the Landfeldt letter largely relate to evidence outside of the period studied in Ryder et al. [1] (2011 and June 2015) and so may be reflective of more current research but not that reviewed. Comments made by Ryder et al. [1] are reflective of those made by Lim [20] which indicated good to moderate agreement between parent proxy and self-assessment.

## Quality assessment / reporting guidelines

STROBE was used and details are provided as to how this assessment was conducted. STROBE is not a quality assessment tool, but more accurately, a set of reporting guidelines. We have modified them and used them as a checklist to assess quality in the absence of something more suitable. At the time of the review we did not know of a better tool.

## Conclusions

The letter by Landfeldt suggests there are shortcomings in the search strategy but then fails to substantiate what these are. Most of the criticism seems to relate to the fact that evidence outside of the time period of review is different to that within the period of review. This may be a very valid observation but unreasonably used as a criticism of the review by Ryder et al. [1].
